# *Entamoeba gingivalis* is associated with periodontal conditions in Chinese young patients: A cross-sectional study

**DOI:** 10.3389/fcimb.2022.1020730

**Published:** 2022-10-07

**Authors:** Junwei Jiao, Mengyao Bie, Xin Xu, Dingyu Duan, Yan Li, Yafei Wu, Lei Zhao

**Affiliations:** ^1^ Department of Periodontics, West China Hospital of Stomatology, Sichuan University, Chengdu, China; ^2^ State Key Laboratory of Oral Diseases & National Clinical Research Center for Oral Diseases, Chengdu, China; ^3^ Department of Endodontics, West China Hospital of Stomatology, Sichuan University, Chengdu, China

**Keywords:** cytokines, dental plaque, *Entamoeba gingivalis*, gingival crevicular fluid, microbiota, periodontitis

## Abstract

**Background:**

This study investigated the prevalence and relative abundance of *Entamoeba gingivalis* (*E. gingivalis*) in Chinese young patients with different periodontal conditions, and its association with subgingival microbial composition, periodontal parameters, and cytokines in gingival crevicular fluid.

**Methods:**

Participants (age: 18–45 years) diagnosed with stage II–IV periodontitis, gingivitis, or periodontal health underwent periodontal examination and sampling. Subgingival plaque was analyzed by 16S+18S sequencing for *E. gingivalis* detection and microbial analysis. The distribution of *E. gingivalis* in subgingival plaque was illustrated by fluorescence *in situ* hybridization. Interleukin-1β, interleukin-8, and tumor necrosis factor-α in gingival crevicular fluid were measured by multiplexed flow cytometric assay.

**Results:**

This cross-sectional study included 120 sites from 60 participants. The prevalence and relative abundance of *E. gingivalis* were significantly increased in periodontitis (p<0.05). The sites were classified into three subgroups according to the relative abundance of *E. gingivalis*: negative group (Eg0, n=56); low-abundance group (Eg1, n=32); and high-abundance group (Eg2, n=32). The subgingival microflora in the subgroups showed stepwise changes at both the phylum and genus levels. The microflora compositions were significantly altered from Eg0 to Eg2 (p<0.001). Co-occurrence network analysis showed that *Porphyromonas*, *Treponema*, *Tannerella*, *Filifactor*, *TG5*, and *Desulfobulbus* were highly correlated with *E. gingivalis* (r>0.6, p<0.001). Correlation analysis showed that *E. gingivalis* was closely associated with important periodontal parameters and cytokines (p<0.01).

**Conclusion:**

*E. gingivalis* was enriched in periodontitis and closely associated with subgingival microbial dysbiosis, periodontal parameters and cytokines in gingival crevicular fluid. Thus, it may be an important pathogen in periodontal disease.

## Introduction

Periodontitis is a chronic inflammatory disease resulting from complex interactions between the biofilm and host inflammatory immune response. The transition from periodontal health to periodontitis is associated with evolution of a health-promoting biofilm to dysbiosis (Meyle and Chapple, 2015). Besides bacteria, viruses, protozoa, fungi, and archaea are also components of the human oral microbiome (Wade, 2013). For example, herpesvirus-bacteria coinfection and their synergistic interaction contributing to periodontal disease has been discussed (Slots, 2015; Chen et al., 2020). The common fungal resident *Candida* has also been investigated in terms of its interactions with other microbiome members and the mediation of dysbiosis (Koo et al., 2018; Diaz and Valm, 2020).

As a parasitic protozoon, *Entamoeba gingivalis* (*E. gingivalis*) is the first amoeba found in humans. Although *E. gingivalis* was frequently detected in periodontitis (particularly advanced periodontitis) during the past several decades (Wantland and Wantland, 1960; Gottlieb and Miller, 1971; Linke et al., 1989), its pathogenicity has not been confirmed. Of note, early studies were based on the microscopic detection of *E. gingivalis*, increasing the risk of its misidentification as a macrophage (Dao et al., 1983). The development of gene amplification by polymerase chain reaction (PCR) offered markedly higher sensitivity and specificity for the detection of *E. gingivalis* (Bonner et al., 2014). In recent years, several PCR-based studies showed that *E. gingivalis* was significantly increased in periodontal pockets of periodontitis. The prevalence in patients with periodontitis and in individuals with periodontal health was 74–88.9% (Zaffino et al., 2019; Bao et al., 2020; Dubar et al., 2020; Yaseen et al., 2021) and 3.3–47.9%, respectively (Bao et al., 2020; Dubar et al., 2020; Yaseen et al., 2021). In a study of the transcriptional activity of periodontal pocket microbiota, *E. gingivalis* had the second highest abundant rRNA found in periodontitis after human rRNA. This accounted for 6.5% of the total RNA reads on average compared with only 0.4% in healthy individuals (Deng et al., 2017). Hence, based on these data and the similarity to its closely-related species *Entamoeba histolytica*, the status of *E. gingivalis* as a potential pathogen contributing to periodontitis has been evaluated (Bonner et al., 2018). The latest *in vitro* study revealed that *E. gingivalis* could invade the inflamed and wounded oral mucosa and further ingest live host cells, thereby showing strong virulence potential (Bao et al., 2020). Further research showed differential expression of genes in *E. gingivalis*-infected gingival epithelial and fibroblast cells, and described different behavior patterns of *E. gingivalis* on the gingival epithelial layer (Bao et al., 2021). These findings indicated that *E. gingivalis* may be an important pathogen in periodontal tissue.

However, the process for defining a specific microorganism as a periodontal pathogen is very complex (Socransky, 1979). Thus far, the role of *E. gingivalis* in the progression of periodontitis is poorly understood. Most studies suggested that *E. gingivalis* has a markedly higher prevalence in periodontitis versus periodontal health; however, quantitative data on this matter are currently scarce. Moreover, some bacteria could reside within *Entamoeba* species and even survive and multiply (Pimenta et al., 2002; Greub and Raoult, 2004). Nevertheless, only few studies focused on the relationship between *E. gingivalis* and the subgingival microbiome, as well as the cytokines in gingival crevicular fluid (GCF). Therefore, the aim of this clinical cross-sectional study was to: (i) investigate the prevalence and relative abundance of *E. gingivalis* in Chinese young patients with different periodontal conditions; (ii) analyze the association between *E. gingivalis* and subgingival microflora, cytokines levels in GCF, and periodontal clinical parameters; and (iii) illustrate its distribution in the subgingival plaque by fluorescence *in situ* hybridization (FISH).

## Materials and methods

### Patient recruitment

The protocol of the present cross-sectional study was approved by the Medical Ethics Committee of West China Hospital of Stomatology, Sichuan University (approval number: WCHSIRB-D-2020-288) and registered in the Chinese Clinical Trial Registry (ChiCTR2000038928). The study was conducted in accordance with the tenets of the Helsinki Declaration, as revised in 2013. All participants provided written informed consent. Patients who visited the Department of Periodontics in West China Hospital of Stomatology, Sichuan University from October 2020 to October 2021 and volunteers with periodontal health were assessed for eligibility. Participants (age: 18–45 years) without systemic diseases were classified into three groups according to the 2018 European Federation of Periodontology/American Academy of Periodontology classification as follows (Chapple et al., 2018; Papapanou et al., 2018). Periodontal health with intact periodontium (H): 1) no probing attachment loss; 2) probing pocket depths ≤3 mm; 3) bleeding on probing <10%; and 4) no radiological bone loss. Gingivitis with intact periodontium (G): 1) no probing attachment loss; 2) probing pocket depths ≤3 mm; 3) bleeding on probing ≥10%; and 4) no radiological bone loss. Periodontitis stage II–IV (P): 1) more than two non-adjacent sites with interdental probing attachment loss ≥3 mm; 2) more than two non-adjacent sites with probing pocket depth ≥5 mm; and 3) radiological bone loss ≥15%.

The exclusion criteria were: 1) antibiotic therapy or periodontal treatment in the last 6 months; 2) use of other drugs affecting periodontal tissue, such as phenytoin sodium, ciclosporin, nifedipine, verapamil, and diphosphonate drugs; 3) ongoing orthodontic treatment; 4) presence of mental disease; and 5) previous head and neck radiotherapy or chemotherapy.

### Clinical evaluation and sample collection

All clinical evaluations and sample collection were performed by one trained specialist dentist. Parameters, including probing depth (PD), clinical attachment loss (CAL), sulcus bleeding index (SBI), plaque index (Loe and Silness, 1963), and mobility were assessed prior to sampling with a University of North Carolina (UNC) periodontal probe (15 mm). Two different quadrant tooth sites in each participant were selected for sampling: (i) two sites with PD ≤3 mm and no bleeding on probing for group (H); (ii) two sites with PD ≤3 mm and the highest SBI for group (G); and (iii) two sites with the deepest PD for group (P). Tooth sites with periodontal abscess, endo-periodontal lesion, caries, and prosthesis were excluded. Prior to sampling, supragingival plaque was removed using a sterile instrument. After isolation of the selected tooth site with a cotton pellet and gentle air-drying, one 30# absorbent paper point was placed into the pocket or sulcus until mild resistance was sensed, and was left for 30 s to collect GCF. Blood-contaminated samples were discarded. Subgingival plaque was collected from the same site with a sterile Grace curette. All samples were immediately transferred to microcentrifuge tubes and stored at –80°C until analysis.

### DNA extraction and Illumina sequencing

Total genomic DNA was extracted from the subgingival plaque samples using the OMEGA Soil DNA Kit (Omega Bio-Tek, Norcross, GA, USA) according to the instructions provided by the manufacturer. The quantity and quality of extracted DNA were measured using a NanoDrop^®^ ND-1000 spectrophotometer (Thermo Fisher Scientific, Waltham, MA, USA) and agarose gel electrophoresis, respectively. PCR amplification of the V3–V4 region of bacterial 16S rRNA genes was performed using the primers 338F (5’-ACTCCTACGGGAGGCAGCA-3’) and 806R (5’-GGACTACHVGGGTWTCTAAT-3’) (Caporaso et al., 2011). For the detection of protozoa, primers Euk1391F (5′-GTACACACCGCCCGTC-3′) and EukBR (5′-TGATCCTTCTGCAGGTTCACCTAC-3′) were used to target the V9 region of 18S rRNA genes (Maritz et al., 2017). PCR amplicons were purified with Vazyme VAHTS™ DNA Clean Beads (Vazyme, Nanjing, China) and quantified using the Quant-iT PicoGreen^®^ dsDNA Assay Kit (Invitrogen, Carlsbad, CA, USA). After the individual quantification step, amplicons were pooled in equal amounts, and pair-end 2×250 bp sequencing was performed using the Illumina MiSeq platform by Shanghai Personal Biotechnology Co., Ltd (Shanghai, China). Microbiome bioinformatics analysis was performed using QIIME2 2019.4 with slight modification versus the official tutorials (Bolyen et al., 2019). Briefly, raw sequence data were demultiplexed using the demux plugin. Subsequently, the DADA2 plugin was used for quality filtering, denoising, merging, and chimera removal from the sequences. Taxonomy was assigned to amplicon sequence variants (ASVs) using the classify-sklearn naΪve Bayes taxonomy classifier in the feature-classifier plugin against the Greengenes or SILVA Release 132 database for bacteria and eukaryotic microorganisms, respectively.

### Measurement of cytokines in GCF

GCF samples were eluted from the absorbent paper points using assay buffer (100 μL) and centrifugation. The levels of interleukin (IL)-1β, IL-8, and tumor necrosis factor-α (TNF-α) in GCF were measured with the multiplexed flow cytometric assay using a human cytokine kit (HSTCMAG-28SK Kit, Millipore, Billerica, MA, USA) on a Luminex^®^ system (Luminex MAGPIX^®^ with xPONENT, Luminex, USA) according to the instructions provided by the manufacturers. Data were analyzed with software (Milliplex Analyst^®^ 5.1, Merck, Darmstadt, Germany), standard curves, and five-parameter logistic function. Based on the standard curves, the coefficient of variation was calculated and did not exceed 20%. The sensitivity of the Milliplex assay for these three cytokines ranged 0.1–0.24 pg/mL. Out-of-range values were designated as the lowest detectable value. The levels of IL-1β, IL-8, and TNF-α in GCF were calculated and expressed as total amounts (pg) per 30s of sampling time. All samples were analyzed in duplicates.

### FISH on subgingival plaque

Another subgingival plaque sample from one of the patients in group (P) was collected as described above, immersed in dehydrated ethanol (500 μL), and stored at 4°C for FISH. EUB338 (5’-GCTGCCTCCCGTAGGAGT-3’) labeled with Cy3 at both ends for the entire bacterial population (Amann et al., 1990) and *E. gingivalis* FISH probe (5’-TTACTAGAATAGGCGCATTTCGAACAGG-3’) labeled with fluorescein isothiocyanate at both ends were synthesized commercially (Future Biotech, Beijing, China). Plaque material (20 μL) was placed onto a slide coated with poly-L-lysine, baked at 72°C for 2 h, and washed twice with phosphate-buffered saline 5 min. Two different probes were diluted with the hybridization buffer at a ratio of 1:1:50. Following incubation and lysozyme digestion, the specimen was hybridized with probes in a hybridization chamber at 37°C overnight. The images were captured and analyzed by a Nikon Eclipse 80i microscope (Nikon, Tokyo, Japan). The excitation lasers for two probes were used at 488 nm and 555 nm wavelength, respectively.

### Statistical analysis

The sample size was determined based on the variation in the prevalence of *E. gingivalis* in patients with different periodontal conditions. According to previous results (Zaffino et al., 2019; Bao et al., 2020; Dubar et al., 2020; Yaseen et al., 2021), the estimated prevalence of *E. gingivalis* was 15%, 50%, and 85% in group (H), (G), and (P), respectively. It was calculated that at least 16 patients per group were required for 95% power to detect an effect size of 0.5715 with a significance level of 0.05 using PASS 15 (NCSS, Kaysville, Utah, USA). Thus, considering the potential dropout, we decided to include 20 patients in each group.

Sequence data analyses were mainly performed using the QIIME2 and R packages (v3.2.0). ASV-level alpha diversity indices, such as Chao1 richness estimator, Shannon diversity index, and Simpson index, were calculated using the ASV table in QIIME2. Beta diversity analysis was performed to investigate the structural variation of microbial communities across samples using Weighted UniFrac distance and visualized *via* principal coordinate analysis. The significance of differentiation in microbiota structure among groups was assessed by permutational multivariate analysis of variance (McArdle and Anderson, 2001). Co-occurrence network analysis was performed by SparCC analysis, and the pseudocount value was set to 10^−6^. A network was constructed, with nodes representing amplicon sequence variants and edges representing correlations.

Chi-squared or Fisher’s exact tests were performed to determine the difference in *E. gingivalis* frequency and other categorical variables among groups. The distributions of ages, clinical periodontal measures, cytokine levels, and relative abundance of microbial taxa were positively skewed. Significant differences between groups were determined using the Kruskal–Wallis test. The associations between different variables were examined using the Spearman correlation test. All statistical analyses were carried out with SPSS 26.0 and GraphPad Prism 9.0. The level of statistical significance was set at p<0.05.

## Results

### Participants

A total of 60 participants (n=20 per group) were enrolled in this study, and 120 sites were analyzed. Demographics and periodontal parameters of sampling sites are shown in **Table 1**. The age of the participants ranged from 20 to 45 years, and most of them were non-smokers, except two and three patients in group (P) and group (G), respectively. On average, 75,735 non-singleton reads and 1,073 ASVs were detected in each sample by 16S sequencing. Moreover, after filtering out the mammalian information, 20,910 non-singleton reads and 32 ASVs were detected in each sample on average by 18S sequencing.

**Table 1:** Patient characteristics and periodontal parameters of sampling sites.

Characteristic	Group (H)(20 patients, 40 sites)	Group (G)(20 patients, 40 sites)	Group (P)(20 patients, 40 sites)	p value
**Age (years)**	24.75 ± 4.58	26.40 ± 4.98	34.25 ± 5.93^*†^	<0.01
**Male sex**	7 (35%)	11 (55%)	4 (20%)	0.084
**Smoker**	—	3 (15%)	2 (10%)	0.353
**PD (mm)**	2.30 ± 0.56	2.85 ± 0.48^*^	7.50 ± 1.65^*†^	<0.05
**CAL (mm)**	—	—	4.97 ± 2.55^*†^	<0.001
**SBI**	0.03 ± 0.16	2.78 ± 0.70^*^	2.93 ± 0.76^*^	<0.001
**PLI**	0.25 ± 0.49	1.73 ± 0.85^*^	1.43 ± 1.17^*^	<0.001
**Mobility**	—	—	0.53 ± 0.75^*†^	<0.001

CAL, clinical attachment loss; PD, probing depth; PLI, plaque index; SBI, sulcus bleeding index; Group (H), periodontal health; Group (G), gingivitis; Group (P), periodontitis with stage II–IV. Continuous variables are represented by the mean ± standard deviation and the determined by Kruskal–Wallis test. Categorical variables are determined by the chi-squared or Fisher’s exact test.

^*^Significant difference versus group H.

^†^Significant difference versus group G.

### Prevalence and relative abundance of *E. gingivalis* in subgingival plaque

**Figure 1:**
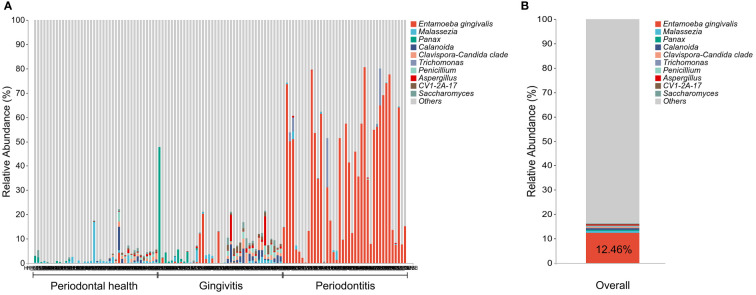
The prevalence and relative abundance of *Entamoeba gingivalis* (*E. gingivalis*) at the genus level. **(A)** The prevalence and relative abundance of *E. gingivalis* at the genus level among different periodontal condition. The top 10 most abundant genera are exhibited. **(B)** The relative abundance of *E. gingivalis* at the genus level in all samples. The top 10 most abundant genera are exhibited.

The prevalence and relative abundance of *E. gingivalis* at the genus level is shown in **Figure 1**. All 40 sites (100%) from 20 patients (100%) in group (P) were *E. gingivalis*-positive versus 21 sites (52.5%) from 13 patients (65%) in group (G) and three sites (7.5%) from three patients (15%) in group (H) (p<0.05). The variation in the relative abundance of *E. gingivalis* was also statistically significant, with 35.93%, 1.34%, and 0.11% recorded in group (P), (G), and (H), respectively (**Table 2**). Subsequently, all 120 sites were classified into three subgroups according to the relative abundance of *E. gingivalis* for further analysis: negative group (Eg0, n=56); low-abundance group (Eg1, relative abundance <10%, n=32); and high-abundance group (Eg2, relative abundance ≥10%, n=32).

**Table 2:** Comparison of the prevalence and relative abundance of *Entamoeba gingivalis* (*E. gingivalis*) at the genus level in patients with different periodontal condition.

	Group (H)		Group (G)		Group (P)		p value
	subjects (%)	sites (%)	subjects (%)	sites (%)	subjects (%)	sites (%)	
***E. gingivalis* positivity**	3 (15%)	3 (7.5%)	13 (65%) *	21 (52.5%) *	20 (100%) * †	40 (100%) * †	<0.05
***E. gingivalis* relative abundance**	0.11%		1.34%*		35.93%* †		<0.05

Group (H), periodontal health; Group (G), gingivitis; Group (P), periodontitis with stage II–IV.

Data are determined by the chi-square or fisher exact test.

*Significant difference versus group H.

^†^Significant difference versus group G.

### Association between *E. gingivalis* and subgingival microbial composition

**Figure 2:**
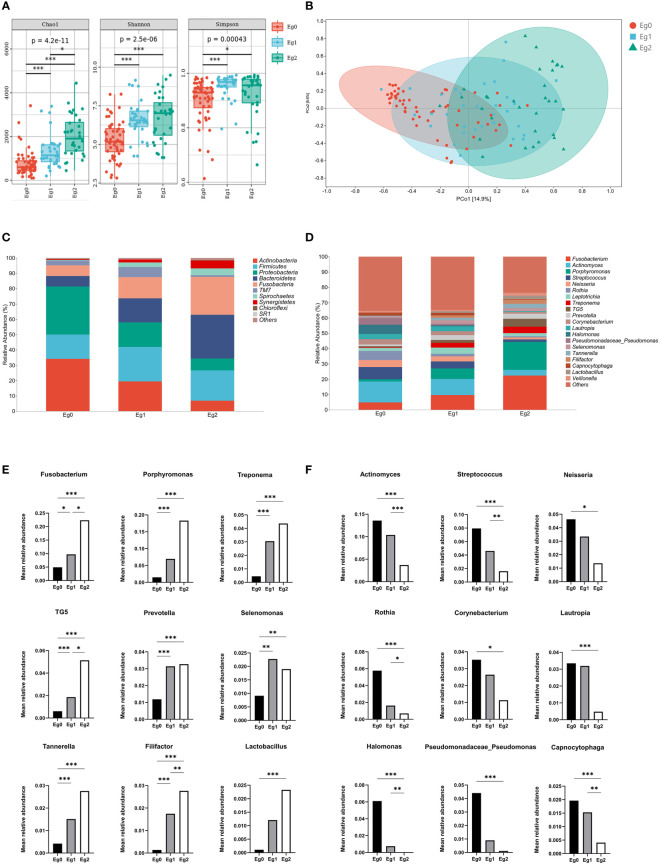
Alterations in bacterial diversity and community structure among *Entamoeba gingivalis* (*E. gingivalis*) subgroups. **(A)** Comparison of bacterial alpha diversity by Chao1, Shannon index, and Simpson index among the *E. gingivalis* subgroups. **(B)** Structural discrepancy in microbial communities by principal coordinate analysis based on Weighted UniFrac distance among the *E. gingivalis* subgroups. The 95% confidence ellipses show the separation of microbial communities among the *E. gingivalis* subgroups. **(C)** Barplot showing the bacterial proportion at the phylum level among the *E. gingivalis* subgroups. The top 10 most abundant phyla are exhibited. **(D)** Barplot showing the bacterial proportion at the genus level among the *E. gingivalis* subgroups. The top 20 most abundant genera are exhibited. **(E, F)** Significantly increased and decreased bacteria of the top 20 abundant genera among the *E. gingivalis* subgroups. Eg0, *E. gingivalis* negative group (n=56). Eg1, *E. gingivalis* low-abundance group (relative abundance <10%, n = 32). Eg2, *E. gingivalis* high-abundance group (relative abundance ≥10%, n=32). Data are represented by the mean relative abundance and determined by the Kruskal–Wallis test. *p < 0.05, **p < 0.01, ***p < 0.001.

The comparison of bacteria alpha diversity among the *E. gingivalis* subgroups is shown in **Figure 2A**. Bacterial richness by Chao1 was significantly increased from Eg0 to Eg2 (p<0.001). The diversity determined by the Shannon and Simpson indices in Eg1 and Eg2 were significantly higher than that noted in Eg0 (p<0.05); of note, the differences between Eg1 and Eg2 were not significant. The principal coordinate analysis based on Weighted UniFrac distance showed a tendency for clustering of the bacterial microbiome in different subgroups and apart from each other (**Figure 2B**). Moreover, permutational multivariate analysis of variance analysis demonstrated that the bacterial community structures were significantly different from Eg0 to Eg2 (p<0.001).

The subgingival taxonomic composition in the *E. gingivalis* subgroups showed stepwise changes at both the phylum (**Figure 2C**) and genus levels (**Figure 2D**). Among the top 20 most abundant bacteria genera (relative abundance ≥1%), nine were significantly increased (**Figure 2E**) and nine were decreased (**Figure 2F**) from Eg0 to Eg2 (p<0.05). These results suggested that the subgingival microbial composition was significantly altered among the *E. gingivalis* subgroups.

**Figure 3:**
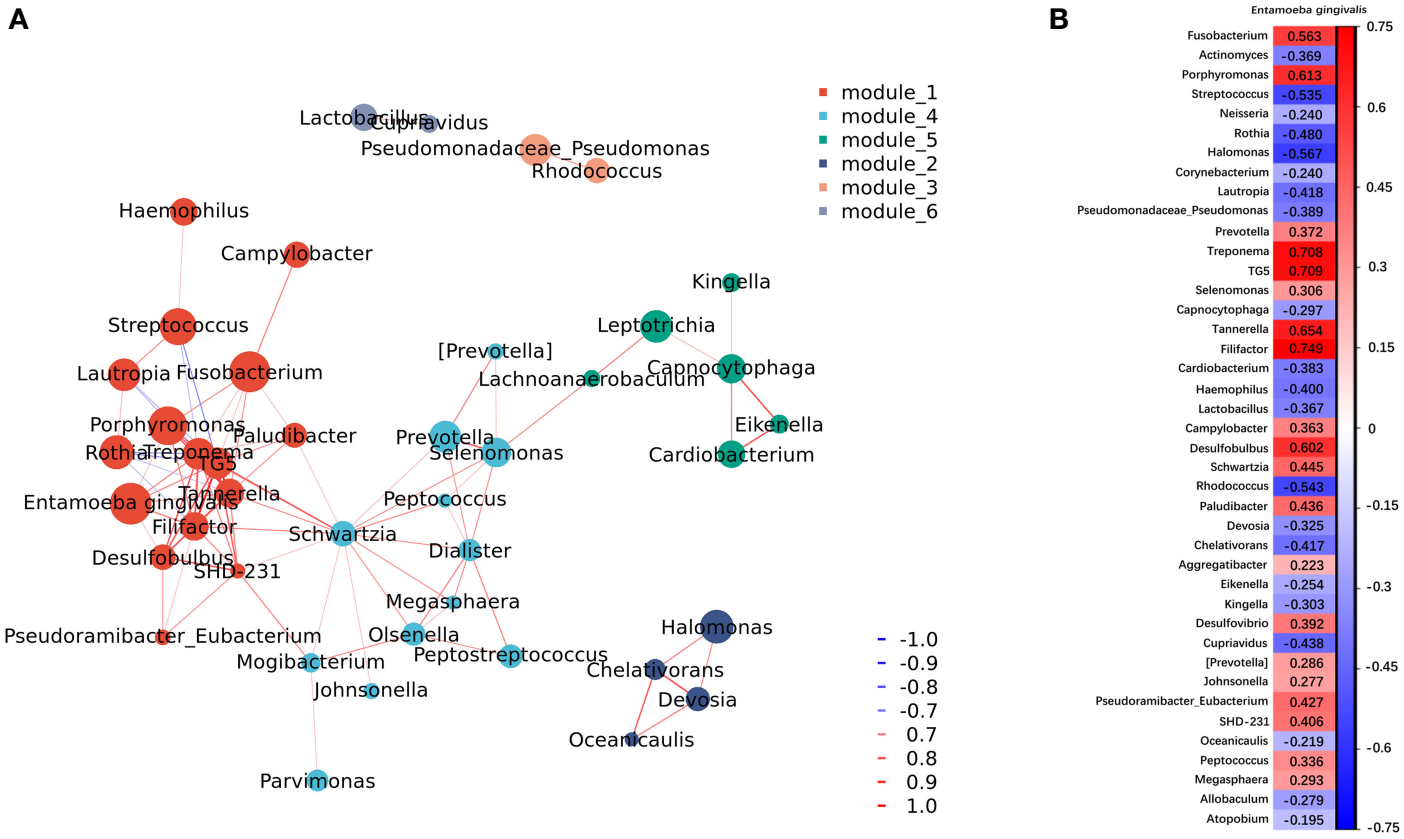
Co-occurrence network analysis between microorganisms at the genus level. **(A)** Network of co-occurrence showing the correlation between *Entamoeba gingivalis* (*E. gingivalis*) and the top 50 abundant bacteria genera with a coefficient |r|>0.6. Six modules were classified according to the intensity of their relationship. Each genus is colored according to the module it belongs to. The color intensity of connecting lines reflects the correlation coefficient, while the node size reflects the abundance. Red and blue lines represent positive and negative correlations, respectively. **(B)** The results of the co-occurrence network analysis between *E. gingivalis* and the top 50 abundant bacteria genera with a p < 0.05. The color intensity of grids reflects the strength of the correlation. Red and blue represent positive and negative correlations, respectively. The numbers are the correlation coefficients.

Co-occurrence network analysis showed the correlation between *E. gingivalis* and bacteria at the genus level (**Figure 3A**). Of the top 50 bacteria genera in abundance, 41 were significantly associated with *E. gingivalis* (p<0.05) (**Figure 3B**). Six genera, including the red complex organisms genera *Porphyromonas*, *Treponema*, and *Tannerella* (Socransky et al., 1998) as well as *Filifactor*, *TG5*, and *Desulfobulbus*, were highly correlated with *E. gingivalis* (coefficient: >0.6; p<0.001).

### Association between *E. gingivalis* and periodontal parameters

**Figure 4:**
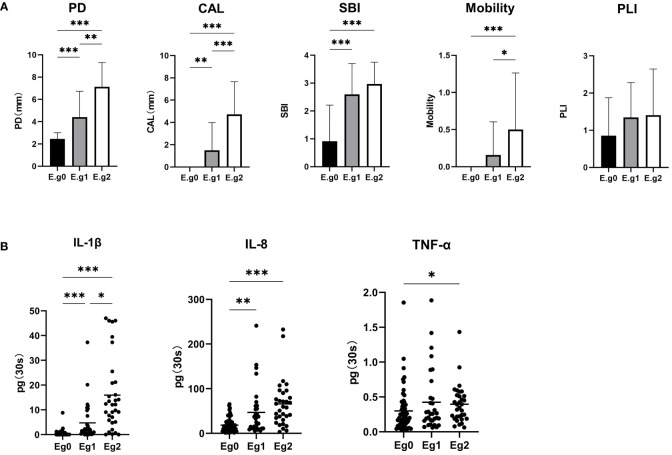
Alterations in clinical periodontal parameters and cytokines in gingival crevicular fluid **(GCF)** among the *Entamoeba gingivalis* (*E. gingivalis*) subgroups. **(A)** Comparison of clinical parameters among the *E. gingivalis* subgroups. CAL, clinical attachment loss (mm); PD, probing depth (mm); PLI, plaque index; SBI, sulcus bleeding index. Data are represented by the mean ± standard deviation. **(B)** Comparison of cytokines in GCF among the *E. gingivalis* subgroups. Eg0, *E. gingivalis* negative group (n=56). Eg1, *E. gingivalis* low-abundance group (relative abundance <10%, n = 32). Eg2, *E. gingivalis* high-abundance group (relative abundance ≥10%, n=32). Data are represented by the total amount (pg/30s) in GCF. The stub lines denote the mean. All data were determined by the Kruskal–Wallis test. *p < 0.05, **p < 0.01, ***p < 0.001.

The periodontal clinical parameters among *E. gingivalis* subgroups are shown in **Figure 4A**. PD and CAL were significantly increased from Eg0 to Eg2 (p<0.01). The SBI was higher in Eg1 and Eg2 versus Eg0 (p<0.001); notably, there was no significant difference between Eg1 and Eg2. Mobility was higher in Eg2 versus Eg0 and Eg1 (p<0.05), but there was no significant difference between Eg0 and Eg1. Although the plaque index showed an increasing trend from Eg0 to Eg2, the difference was not statistically significant.

Correlation analysis was performed between the relative abundance of *E. gingivalis* and periodontal parameters using the Spearman test. For comparison, the abundance of red complex organisms genera *Porphyromonas*, *Treponema*, *Tannerella* as well as *Fusobacterium* was also analyzed (**Table 3**). *E. gingivalis* was significantly associated with all five parameters (p<0.01). Regarding PD, CAL, and mobility, *E. gingivalis* exhibited the highest r value among these five microorganisms.

**Table 3:** Spearman correlation test between the relative abundance of microorganisms and periodontal parameters, as well as cytokines in gingival crevicular fluid (GCF).

Microorganism	PD (mm)		CAL (mm)		SBI		PLI		Mobility		IL-1β (pg/30s)		IL-8 (pg/30s)		TNF-α (pg/30s)		
	r	p value	r	p value	r	p value	r	p value	r	p value	r	p value	r	p value	r	p value
***E. gingivalis***	0.787^†^	<0.0001	0.815^†^	<0.0001	0.605	<0.0001	0.240	0.0082	0.449^†^	<0.0001	0.750^†^	<0.0001	0.530^†^	<0.0001	0.240^†^	0.0083
***Porphyromonas***	0.556	<0.0001	0.468	<0.0001	0.612	<0.0001	0.335	0.0002	0.354	<0.0001	0.627	<0.0001	0.321	0.0004	0.191	0.0372
***Treponema***	0.592	<0.0001	0.539	<0.0001	0.709^†^	<0.0001	0.381	<0.0001	0.387	<0.0001	0.701	<0.0001	0.398	<0.0001	0.204	0.0256
***Tannerella***	0.571	<0.0001	0.518	<0.0001	0.659	<0.0001	0.437^†^	<0.0001	0.332	0.0002	0.665	<0.0001	0.323	0.0003	ns	
***Fusobacterium***	0.451	<0.0001	0.410	<0.0001	0.460	<0.0001	0.259	0.0043	ns		0.503	<0.0001	ns		ns	

CAL, clinical attachment loss; E. gingivalis, Entamoeba gingivalis; GCF, gingival crevicular fluid; IL-1β, interleukin-1β; IL-8, interleukin-8; PD, probing depth; PLI, plaque index; SBI, sulcus bleeding index; TNF-α, tumor necrosis factor-α; ns, no significance (p>0.05).

^†^Highest r value among these five microorganisms.

### Association between *E. gingivalis* and cytokines in GCF

The total amounts (pg/30s) of IL-1β, IL-8, and TNF-α in GCF among *E. gingivalis* subgroups are presented in **Figure 4B**. The levels of IL-1β were significantly increased from Eg0 to Eg2 (p<0.05). The levels of IL-8 were higher in Eg1 and Eg2 versus Eg0 (p<0.01); however, the difference between these two subgroups was not statistically significant. TNF-α also showed an increasing trend from Eg0 to Eg2; nevertheless, the significant difference only existed in Eg0 versus Eg2 (p<0.05). Similar to periodontal parameters, a correlation analysis was performed for the cytokines and the five microorganisms (i.e., *E. gingivalis*, *Porphyromonas*, *Treponema*, *Tannerella*, *Fusobacterium*). The results showed that *E. gingivalis* was significantly associated with the three cytokines (p<0.01) and had the highest r value (**Table 3**).

### Distribution of *E. gingivalis* in subgingival plaque

**Figure 5:**
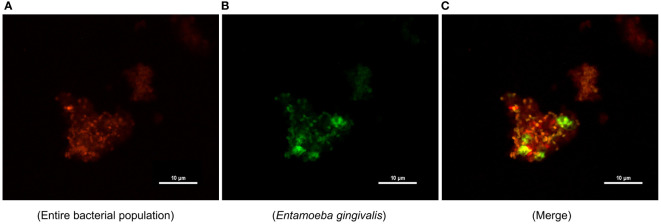
Distribution of *Entamoeba gingivalis* (*E. gingivalis*) in subgingival plaque detected by fluorescence *in situ* hybridization. **(A)** The entire bacterial population is shown in red. **(B)** *E. gingivalis* is shown in green. **(C)** Merging of *E. gingivalis* and the entire bacterial population. Scale bar: 10 μm.

The images of FISH-stained microorganisms in subgingival plaque are presented in **Figure 5**. The entire bacterial population and *E. gingivalis* are shown in red and green (**Figures 5A, B**), respectively; and the co-occurrence was merged in **Figure 5C**. The distribution of *E. gingivalis* was generally consistent with that of the bacterial population, while its signal intensity was lower.

## Discussion

To our knowledge, this cross-sectional study is the first to simultaneously detect both *E. gingivalis* and the bacterial microbiome in subgingival plaque by 16S+18S rRNA gene sequencing. A previous study using a similar approach revealed that 31% of the participants were positive for *E. gingivalis* (Stensvold et al., 2021). However, the samples used in that analysis were oral washings, and periodontal examinations were not conducted. In the present study, our data showed that 60% of the 60 Chinese young participants harbored *E. gingivalis* in subgingival plaque, including 15% in group (H), 65% in group (G), and 100% in group (P). This variation was consistent with that observed in previous studies (Wantland and Wantland, 1960; Bao et al., 2020; Dubar et al., 2020). It is noteworthy that all 40 sites in patients with periodontitis were positive for *E. gingivalis*; this prevalence (100%) was the highest among rates reported thus far by PCR-based studies (Bonner et al., 2014; Garcia et al., 2018; Zaffino et al., 2019; Bao et al., 2020; Dubar et al., 2020; Yaseen et al., 2021). This discrepancy between results may be attributed to differences between races, regions, sampling protocols, and detection methods. In addition, for our analysis, we selected tooth sites with the deepest PD that could be more representative of the severity of periodontitis. The mean PD and CAL values of sampling sites in the periodontitis group were 7.50 mm and 4.97 mm, respectively. These findings indicated that the severity of periodontitis in the present study was close to stage III–IV. Apart from the higher prevalence of *E. gingivalis* in periodontal disease, a significant increase in relative abundance from periodontal health to periodontitis was also recorded. Furthermore, it was the most abundant among the eukaryotic genera examined in the present study, with an average abundance of 12.46% (**Figure 1**). Thus, these data may explain the previous observation that *E. gingivalis* had the second most abundant rRNA found in periodontitis after that of the human host (Deng et al., 2017).

It is clear that the development of periodontitis is accompanied by profound alterations in the composition of subgingival microbial communities (Curtis et al., 2020). Our results revealed that the richness and evenness of the subgingival microbiome were significantly increased among the *E. gingivalis* subgroups. This observation was in accord with the results obtained through analysis of oral washing samples (Stensvold et al., 2021). These changes also occur during the transformation from periodontal health to periodontitis (Griffen et al., 2012; Abusleme et al., 2013; Camelo-Castillo et al., 2015). Moreover, it has been shown that the subgingival microbial communities are distinctly separate in periodontal health, gingivitis, and periodontitis (Abusleme et al., 2021). In this study, the microbial communities in the subgroups were gradually separated as the relative abundance of *E. gingivalis* increased. Nevertheless, this difference was not observed in the analysis of oral washing samples (Stensvold et al., 2021). A previous study identified 13 genera as dominant bacteria, with the relative abundance accounting for >80% of the microbial community in subgingival plaque (Mark Welch et al., 2016). Almost all of them were included in the top 20 abundant genera in the present study (except for *Haemophilus*, which ranked 21st). Among these 20 genera, eight of nine significantly increased genera from Eg0 to Eg1 were associated with periodontitis (i.e., *Fusobacterium*, *Porphyromonas*, *Prevotella*, *Treponema*, *TG5*, *Selenomonas*, *Tannerella*, and *Filifactor*) (Curtis et al., 2020; Abusleme et al., 2021; Cai et al., 2021). Moreover, seven of nine decreased genera were associated with periodontal health, namely *Actinomyces*, *Streptococcus*, *Neisseria*, *Rothia*, *Corynebacterium*, *Lautropia*, and *Capnocytophaga* (Curtis et al., 2020; Abusleme et al., 2021; Cai et al., 2021). A previous study has shown that the same changes in *Treponema*, *Filifactor*, and *Actinomyces* by *E. gingivalis* occurred in saliva (Stensvold et al., 2021). Further correlation network analysis identified *Porphyromonas*, *Treponema*, *Tannerella*, *Filifactor*, *TG5*, and *Desulfobulbus* (also defined as a periodontitis biomarker (Curtis et al., 2020; Cai et al., 2021) as strongly associated genera with *E. gingivalis*, with a coefficient > 0.6. These results indicated that *E. gingivalis* is closely associated with dysbiosis of the subgingival microbiome and several important periodontal pathogens. Furthermore, the associations may be stronger in the subgingival environment than in saliva. However, the mechanism of the interaction between *E. gingivalis* and periodontal pathogens is still poorly understood. On the one hand, although bacteria might be one of the food sources of amoeba, some of them could still survive and even multiply after phagocytosis (Pimenta et al., 2002; Greub and Raoult, 2004). As discussed in previous study, some periodontal pathogens might be sheltered within *E. gingivalis* and transferred to other periodontal tissues with its movement (Trim et al., 2011). On the other hand, study *in vitro* revealed that *E. gingivalis* could only invade into periodontal tissue after the epithelium was slightly punctured but not in intact tissue (Bao et al., 2020). Thus, *E. gingivalis* in periodontal pockets could benefit from the destruction of the epithelium caused by periodontal pathogens, which leads to its further invasion of gingival tissue.

Periodontitis is a chronic systemic inflammatory disease, and neutrophils play a pivotal role in early lesions (Kurgan and Kantarci, 2018), while inflammatory cytokines (e.g., IL-1β and TNF-α) are crucial in the pathogenesis (Xiao et al., 2021). The levels of cytokines in GCF are common material to reflect the inflammatory state of periodontal tissues. Different analytical techniques and reporting of data have been discussed (Wassall and Preshaw, 2016). In the present study, the levels of cytokines in GCF were expressed as the total amount per 30s of sampling time, as previously described (Ye et al., 2020; Arvikar et al., 2021). Previous *in vitro* studies showed that the expression of the neutrophil chemokine *IL8* gene, as well as that of *IL1β* and *TNF*, was significantly increased in *E. gingivalis*-infected gingival epithelial cells (Bao et al., 2020; Bao et al., 2021). Similar results were revealed from our GCF data. The increase of IL-8 may explain the observation that *E. gingivalis* was often surrounding by abundant neutrophils (Bhaijee and Bell, 2011). Furthermore, *E. gingivalis* could trigger signals leading to modifications in the nuclear and cytoplasmic morphology of these cells, thereby inhibiting the function of neutrophils as the first line of defense. These processes might be linked to proteolytic activity of *E. gingivalis*, or to the effect on NETosis (Bonner et al., 2018). Among pathogens linked to periodontitis, *Porphyromonas gingivalis* (*P. gingivalis*) is well-documented and recognized as a predominant contributor (Hajishengallis, 2009). Interestingly, while both *E. gingivalis* and *P. gingivalis* inhibited the proliferation of primary gingival epithelial cells, the increase in the expression of *IL8* and *IL1β* was higher in *E. gingivalis*-infected cells than in *P. gingivalis*-infected cells (Bao et al., 2020). In this study, *E. gingivalis* was the microorganism that demonstrated the strongest association with the three aforementioned cytokines, as well as PD, CAL, and mobility, thereby exhibiting its strong virulence potential in periodontal tissue.

By performing FISH on subgingival plaque samples, we visualized the amount of *E. gingivalis* at the background of the entire bacterial population. However, the microbial distribution should be treated with caution because the structure of the subgingival plaque could be disrupted during curettage and deposition. The two subtypes of *E. gingivalis* (i.e., ST1 and ST2) and their different prevalence rates have been described (Garcia et al., 2018; García et al., 2018). Another technical limitation of the present study is that the sequencing data failed to recognize these two subtypes, and the microbiological analysis was limited to the genus level.

It should be emphasized that determining microorganisms as microbial etiologic agents is a complex logical question. A list of criteria has been suggested, in which lack of association with disease or failure to stop the progression of disease by suppressing the putative pathogen is important (Socransky, 1979). With this in mind, our data revealed an enrichment of *E. gingivalis* in pathological sites and markedly lower levels in healthy sites of *E. gingivalis* in Chinese young patients. Also, they indicated close associations between *E. gingivalis* and subgingival microbial dysbiosis, periodontal parameters, and cytokines in GCF. However, whether *E. gingivalis* is responsible for the progression of periodontitis or merely a biomarker of disease remains to be clarified. Although it has been indicated that direct contact of *E. gingivalis* to gingival epithelial cells could inhibit cell proliferation, the mechanism of its pathogenicity remains to investigate. Once attached to the gingival epithelial cells, *E. gingivalis* could form long cylindrical structures which termed digipodia, extending into the cytoplasma. Through the digipodia, *E. gingivalis* might secrete material into the target cell or ingest material from the target cell (Bao et al., 2021). Besides direct destruction and invasion in periodontal tissues, the immune response and subgingival microbial dysbiosis induced by *E*. *gingivalis* could also be its potential pathogenic mechanism in periodontal disease. Regarding treatment, metronidazole is commonly used against protozoal infections (Bergquist, 2009). Chlorhexidine has also shown high efficacy in the eradication of *E. gingivalis* (Moroz et al., 2019). However, a limited number of studies revealed contrary results concerning the effects of scaling and root planning on *E. gingivalis* (Rashidi Maybodi et al., 2016; Dubar et al., 2020). Furthermore, the relationship between the eradication of *E. gingivalis* and prognosis of periodontitis is poorly understood. Thus, more well-designed, randomized and controlled trials, as well as reliable animal models of infection with *E. gingivalis*, are warranted.

In conclusion, our data demonstrated that *E. gingivalis* was closely associated with periodontal conditions. Thus, it may be an important pathogen in the progression of periodontal disease. The role of protozoal infection in periodontitis should be thoroughly investigated.

## Data availability statement

The data presented in the study are deposited in the NCBI Sequence Read Archive repository, accession number: PRJNA885264 and PRJNA884825.

## Ethics statement

The studies involving human participants were reviewed and approved by Medical Ethics Committee of West China Hospital of Stomatology, Sichuan University. The patients/participants provided their written informed consent to participate in this study.

## Author contributions

JJ contributed to the design, data acquisition and analyses, and drafted and critically revised the manuscript. MB contributed to the data analyses. XX contributed to the design. DD contributed to the design and critically revised the manuscript. YL and YW critically revised the manuscript. LZ obtained funding for the study, contributed to the conception and design, and critically revised the manuscript. All authors approved the final version of the manuscript.

## Funding

This study was supported by the National Natural Science Foundation of China (grant numbers: 81970944 and 81991502), Key Projects of Sichuan Provincial Department of Science and Technology (grant number: 2020YFSY0008), and a research grant from West China Hospital of Stomatology (grant number: LCYJ2019-4).

## Acknowledgments

We thank Zhibin Han (Personalbio) for his suggestions on the sequencing data analysis.

## Conflict of interest

The authors declare that the research was conducted in the absence of any commercial or financial relationships that could be construed as a potential conflict of interest.

## Publisher’s note

All claims expressed in this article are solely those of the authors and do not necessarily represent those of their affiliated organizations, or those of the publisher, the editors and the reviewers. Any product that may be evaluated in this article, or claim that may be made by its manufacturer, is not guaranteed or endorsed by the publisher.
